# Tumor-Resident T Cells, Associated With Tertiary Lymphoid Structure Maturity, Improve Survival in Patients With Stage III Lung Adenocarcinoma

**DOI:** 10.3389/fimmu.2022.877689

**Published:** 2022-05-19

**Authors:** Hua Zhao, Hao Wang, Yu Zhao, Qian Sun, Xiubao Ren

**Affiliations:** ^1^Department of Immunology, Tianjin Medical University Cancer Institute and Hospital, Tianjin, China; ^2^National Clinical Research Center for Cancer, Tianjin, China; ^3^Key Laboratory of Cancer Prevention and Therapy, Tianjin, China; ^4^Tianjin’s Clinical Research Center for Cancer, Tianjin, China; ^5^Key Laboratory of Cancer Immunology and Biotherapy, Tianjin, China; ^6^Department of Biotherapy, Tianjin Medical University Cancer Institute and Hospital, Tianjin, China

**Keywords:** T_RM_, TLS, B cell, TIL, lung adenocarcinoma

## Abstract

Tertiary lymphoid structure (TLS) and tumor-resident memory T cells (T_RM_) play crucial roles in the anti-tumor immune response, facilitating a good prognosis in patients with cancer. However, there have been no reports on the relationship between T_RM_ and TLS maturity. In this study, we detected T_RM_ and the maturity of TLS by immunofluorescence staining and analyzed the relationship between their distribution and proportion in patients with lung adenocarcinoma (LUAD). The proportion of T_RM_ within TLSs was significantly higher than that outside and was positively correlated with the survival of patients. In addition, the proportions of CD4^+^CD103^+^ T_RM_ and CD8^+^CD103^+^ T_RM_ were significantly increased with the gradually maturation of TLSs. We divided the patients into three levels (grade 1, grade 2, and grade 3) according to the presence of increasing maturation of TLSs. The proportion of CD103^+^ T_RM_ in grade 3 patients was significantly higher than that in grade 1 and grade 2 patients, suggesting a close relationship between CD103^+^ T_RM_ and TLS maturity. Furthermore, positive prognosis was associated with grade 3 patients that exhibited CD103^+^ ${\text{T}}_{\text{RM}}^{\text{High}}$ phenotype.

## Introduction

Immunotherapy, e.g., treatment by immune checkpoint inhibitors (ICIs), has revolutionized therapeutic strategies for treating cancer, including non-small cell lung cancer (NSCLC) (1, 2). Previous studies on the mechanisms of ICIs have largely focused on tumor-infiltrating T cells (3). However, recently the findings of three independent studies have indicated that tertiary lymphoid structures (TLSs) and B cell signatures in the tumor site are key determinants of ICI therapeutic efficacy (4–6).

TLSs are ectopic immune cell aggregates that develop in peripheral tissues in response to a wide range of chronic inflammatory conditions, including tumors (7). The structure of TLSs includes B-cell- and T-cell-enriched areas; they have been reported to be the local site of initiation and maintenance of humoral and cellular immune responses for anti-tumor immunity (8). The activity and function of TLSs differ according to their cellular composition and maturation status. Well-developed TLSs composed of mature dendritic cell (DC)/T cell clusters and CD20^+^ B cell follicles are characterized by the presence of both a CD21^+^ follicular-DC (FDC) network and Ki67^+^ proliferating germinal center (GC)-B cells (9, 10). The density of mature TLS is associated with improved prognosis and is an effective predictive biomarker in cancer patients (11, 12). Researchers can synthesize tumor-specific antibodies, which are considered specific markers for prognosis (6). Moreover, B cells in TLSs can function as antigen-presenting cells and are associated with the induction of cytotoxic T cells (13). Therefore, these structures are major sources of tumor-infiltrating lymphocytes (TILs) and regulate the anti-tumor response (14).

Tissue-resident memory T (T_RM_) cells are tumor antigen-reactive TILs that produce a magnitude of cytotoxic mediators, such as granzyme B and perforin, as well as cytokines, such as interferon-gamma (IFN-γ) and tumor necrosis factor (TNF), in the tumor microenvironment (TME) (15, 16). T_RM_ is a newly discovered subset of long-lived memory T cells that reside permanently in peripheral tissues without recirculation (17). In the tumor tissue, they mediate regional tumor surveillance and exhibit a protected anti-tumor function (15, 18). The permanence of T_RM_ in NSCLC is mainly mediated by the expression of integrin αE (CD103) β7, which binds to E-cadherin in epithelial cells (19, 20). T_RM_ is positively correlated with the survival of patients with cancer, including lung cancer (21, 22). The presence of intra-tumor CD8^+^CD103^+^T_RM_ cells could predict a good clinical response in PD-1/PD-L1 blockade immunotherapy (23). CD8^+^ T_RM_ cells have been mainly located around TLSs—both are associated with a better prognosis in patients with gastric cancer (24). These results indicate that tumor-resident T cells may have a close relationship with TLSs. However, there have been no reports on the association of T_RM_ subset distribution with TLS maturation and their relationship with the prognosis of patients.

Because patients with stage III NSCLC usually have quite heterogeneous prognoses, we selected patients with stage III lung adenocarcinoma (LUAD) for the current study. The aim of this study was to investigate the clinical significance of TLS maturation in patients with LUAD, its association with the spatial distribution of distinct T_RM_ subsets in LUAD.

## Materials and Methods

### Patients and Tumor Specimens

Forty-nine patients with stage III primary LUAD who underwent surgical resection at Tianjin Medical University Cancer Institute and Hospital between January 2015 and May 2016 were enrolled in this retrospective study. Pathological TNM staging was histologically diagnosed based on the 7th edition of the Union for International Cancer Control TNM classification. The inclusion criterion was complete clinical data, standardized postoperative treatment and accurate pathological diagnosis. All patients underwent surgical resection of R0, and adjuvant therapy was mainly platinum-based chemotherapy, supplemented by radiotherapy or targeted therapy, when necessary. The exclusion criteria were those who had received anti-cancer treatment before surgery, had a second primary tumor, or were lacking follow-up. In this study, TLS positive tissues were selected for subsequent experiments which confirmed through hematoxylin and eosin (HE) staining slices. Formalin-fixed paraffin-embedded tumor tissues were collected from 49 patients for subsequent immunohistochemical staining and multiple immunofluorescence staining (**Table 1**). The study was approved by the Ethics Committee of the Tianjin Medical University Cancer Institute and Hospital. All patients signed relevant informed consent forms.

**Table 1:** Baseline characteristics of patients (n=49).

Variable	Population, n (%)
Gender	
Male	24 (49%)
Female	25 (51%)
Age (years)	
<60	28 (57%)
≥60	21 (43%)
T stage	
T_1_	29 (59%)
T_2_+T_3_+T_4_	20 (41%)
N stage	
N_1_+N_2_	43 (88%)
N_3_	6 (12%)
TNM stage	
IIIA	39 (80%)
IIIB	10 (20%)
Micropapillary	
Positive	22 (45%)
Negative	27 (55%)
EGFR mutation	
Positive	15 (60%)
Negative	10 (40%)
Smoking	
Never	26 (53%)
Smoking	23 (47%)

### Multiple Immunofluorescence Staining

Multiple immunofluorescence staining was performed using a PerkinElmer Opal 7-color Technology Kit (NEL81001KT). The tumor specimens in paraffin-embedded blocks were cut into 4-μm-thick sections. The sections were deparaffinized in xylene and rehydrated in ethanol. Microwave repair was performed using EDTA buffer (PH=9.0) for 20 min. After cooling, the tissue was sealed with an antibody blocker at room temperature. The sections were then incubated overnight with primary antibody in a refrigerator at 4°C, and on the second day, the sections were co-incubated with poly-HRP-MS/Rb for 10 min at room temperature. Visualization was performed using Opal TSA (1:100). EDTA buffer was then heated by MWT to remove the AB-TSA complex. These steps were repeated for each round of the multiple staining. TSA-stained sections were washed with MWT and counterstained with DAPI (1:100) for 10 min. Using this staining method, all samples were stained with the primary antibody for CD20 (1:600 dilution, clone L26, Abcam) visualized with Opal520 TSA, CD3 (1:400 dilution, clone SP162, Abcam) visualized with Opal540 TSA, CD103 (1:500 dilution, clone EPR4166(2), Abcam) visualized with Opal570 TSA, Bcl6 (1:200 dilution, clone LN22, Novus) visualized with Opal620 TSA, CD4 (1:1000 dilution, clone EPR6855, Abcam) visualized with Opal650 TSA, CD21(1:800 dilution, clone EP3093, Abcam) visualized with Opal690 TSA. Finally, the sections were covered with an anti-fluorescence attenuating tablet and cover glass.

### Multispectral Imaging and TLS Evaluation

Tumor sections were scanned using a PerkinElmer Mantra Quantitative Pathology Imaging System at 200× magnification. Multispectral images were obtained using PerkinElmer inform Image Analysis software (version 2.4.0). Spectral libraries were built from the images of single-stained tissues with each antibody. The TLSs were then manually distinguished. We collected all TLSs of every tumor section and randomly collected three to five fields from areas outside the TLSs. A total of 958 fields were collected, including 807 TLSs and 151 outside fields of TLS.

The density of TLS was calculated as the number of TLSs per mm^2^ of the tumor region in the sections. Immune subsets were determined by antibody expression, including CD4^+^ T cells, CD8^+^ T lymphocytes (CD3^+^CD4^-^), B cells (CD20^+^), FDC (CD21^+^), CD3^+^ T_RM_ (CD3^+^CD103^+^), CD4^+^ T_RM_ (CD4^+^CD103^+^), CD8^+^ T_RM_ (CD3^+^CD4^-^CD103^+^), and GC reaction (CD20^+^Bcl-6^+^) (25). The proportion of the immune subsets in each TLS (or field) was calculated as the percentage of this subpopulation to all nucleated cells in the TLS (or field). The proportion of the immune subsets in each patient was calculated by the average proportion in all fields (within the TLS and outside the TLS) across the entire section.

### Statistical Analysis

Disease-free survival (DFS) was defined as the time from the date of surgery to tumor recurrence. The surv_cutpoint function in the survminer R package (version 4.1.2) was used to obtain the cutoff value of immune subsets proportion. Then the different immune subsets inside and outside TLS were divided into “high” and “low” groups. Kaplan-Meier curve was drawn with the survminer R package (4.1.2). The log-rank test in survival R package (4.1.2) was used to calculate the P value. Both the survminer and survival R package were downloaded from the public resource website: https://cran.r-project.org/. When comparing the prognostic differences of more than two of sub-groups after combining TLS score and T_RM_, *P* value and HR ratio was calculated with log-rank test in GraphPad Prism software.

Chi-square (and Fisher’s exact) test was used to evaluate the relationship between grade score, CD3^+^ CD103^+^ TRM and clinicopathological features. Wilcoxon rank test (paired nonparametric t test) was used to compare the difference of CD103^+^subsets inside and outside TLS. Kruskal-Wallis *H* test was used to compare the differences of immune subsets proportion among different sub-group. All statistical analyses, except survival analyses, were performed with GraphPad Prism (version 9.1.0, US). *P* values of < 0.05 were considered statistically significant.

## Results

### TLS in Patients With Stage III LUAD

**Figure 1:**
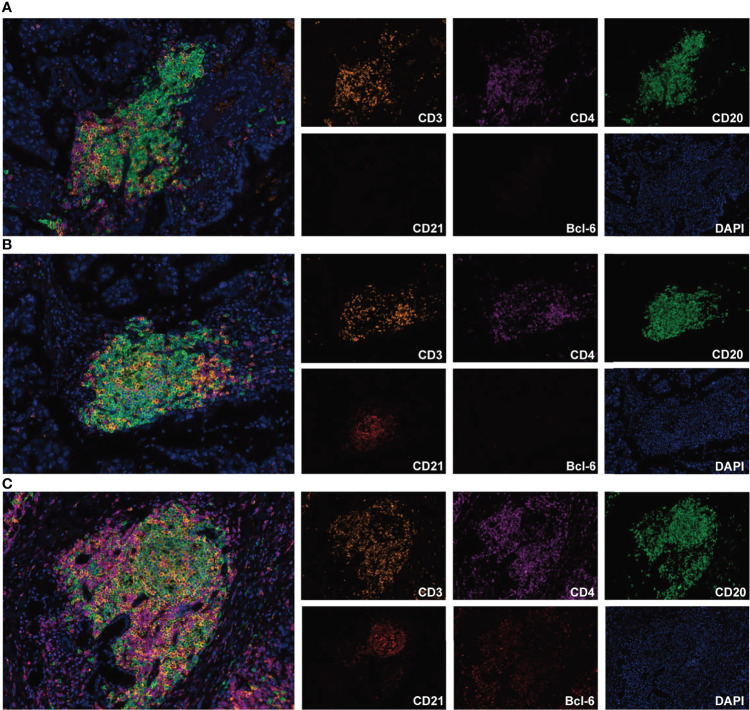
Representative images of TLS maturity (magnification, ×200). The slide was stained with CD3 (orange), CD4 (purple), CD20 (green), CD21 (brown), Bcl-6 (red), and DAPI (blue). **(A)**, E-TLS, both FDC and Bcl-6 markers were negative. **(B)**, PFL-TLS, FDC positive and Bcl-6 negative. **(C)**, SFL-TLS, both FDC and Bcl-6 markers were positive.

**Figure 2:**
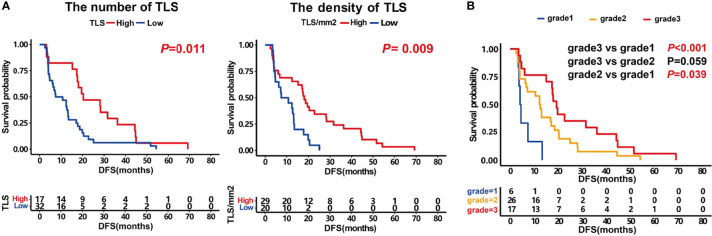
Prognosis impact of the number and density of TLS and patients score. **(A)** Kaplan–Meier survival curves showing DFS according to the number of TLS (*P* =0.011) and the density of TLS (*P* =0.009). **(B)** Kaplan–Meier survival curves showing DFS according to patients score. *P* values were calculated by the log-rank test.

According to the increasing prevalence of FDCs and the maturation of B cells, TLSs were classified into three maturity stages: 1) early TLS (E-TLS), characterized by dense lymphocytic aggregates without CD21 and Bcl-6 expression (**Figure 1A**); 2) primary follicle-like TLS (PFL-TLS), characterized by lymphocytic clusters with central network CD21 expression, but no GC reaction (Bcl-6^-^) (**Figure 1B**); and 3) secondary follicle-like TLS (SFL-TLS), characterized by lymphocytic clusters with GC reaction (CD20^+^Bcl-6^+^) (**Figure 1C**).

For the first time, we divided patients into three levels based on the maturity of TLSs: 1) grade 1: patients with TLSs characterized by only E-TLSs, and without PFL-TLSs and SFL-TLSs; 2) grade 2: patients with TLSs characterized by E-TLSs and at least one PFL-TLS, but no SFL-TLS; and 3) grade 3: patients with TLSs characterized by at least one SFL-TLS in the tumor tissue (**Table 2**).

**Table 2:** Patients score criteria in this study.

Score	E-TLS	PFL-TLS	SFL-TLS
grade 1	+	–	–
grade 2	+	+	–
grade 3	+	+	+

### The Relationship Between TLS and Prognosis

We first evaluated the prognostic impact of the number and density of TLSs in patients. Kaplan–Meier analysis showed that patients with higher numbers of TLSs had a much better prognosis (median DFS 18.7 months vs. 7.4 months, *P* = 0.011, **Figure 2A**, left). A higher density of TLS was also positively associated with a good DFS (median 17.3 months vs. 12.4 months, *P* = 0.009, **Figure 2A**, right).

Furthermore, we analyzed the prognosis of patients with different grades. The results showed that the prognosis of grade 3 patients was significantly higher than that of those in grade 1 (median DFS 19.5 months vs. 4.3 months, *P <*0.001, **Figure 2B**). The grade 2 patients also had a better DFS than those in grade 1 (median 12.6 months vs. 4.3 months, *P* =0.039). The prognosis of grade 3 patients tended to be better than those in grade 2 (median DFS 19.5 months vs. 12.6 months, *P* =0.059, **Figure 2B**). These results indicate that the maturity of TLS is crucial for the prognosis of patients.

### T_RM_^High^ Within TLS Was Associated With Good Prognosis

**Figure 3:**
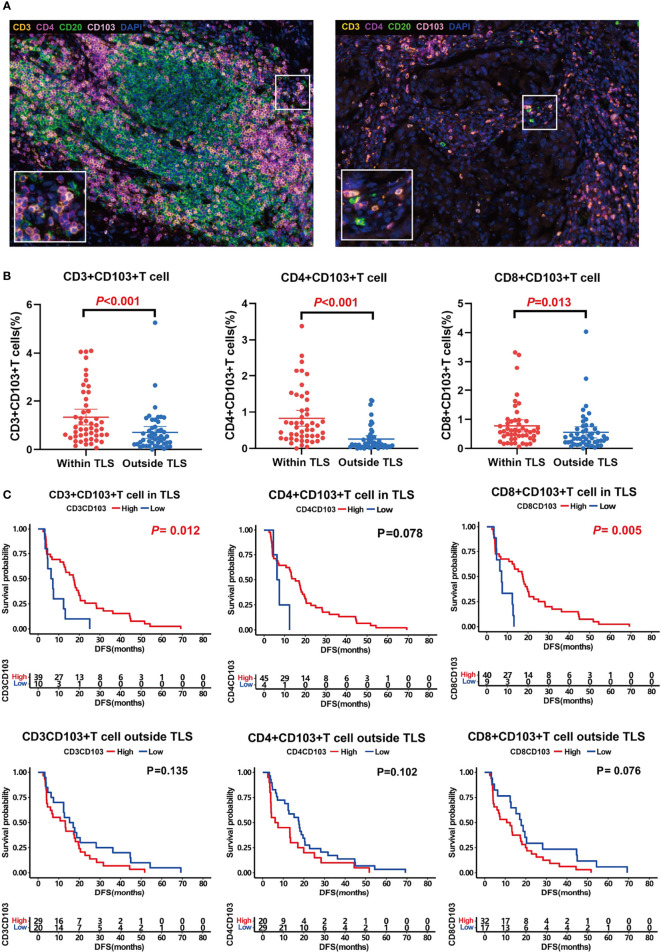
T_RM_ distribution and its association with prognosis. **(A)** Representative images of T_RM_ inside (left) and outside (right) TLSs. **(B)** Comparison of CD3^+^CD103^+^ T_RM_ (left, *P < *0.001), CD4^+^CD103^+^ T_RM_ (middle, *P < *0.001), and CD8^+^CD103^+^ T_RM_ (right, *P < *0.001) distribution inside and outside TLS. T_RM_ were mainly located in TLS. C, Influence of T_RM_ inside and outside of TLS on patient prognosis. T_RM_ inside TLS predicted a better prognosis.

By comparing the difference in the proportion of T_RM_ inside and outside the TLS, we determined that all T_RM_ subsets were mainly located in TLS, especially CD4^+^ T_RM_ (**Figures 3A, B**). The proportion of CD3^+^ T_RM_ in TLS (mean ± SD: 1.34% ± 1.13%) was significantly higher than that outside (mean ± SD: 0.71% ± 0.84%), *P <*0.001. The proportion of CD4^+^ T_RM_ in TLS (mean ± SD: 0.83% ± 0.75%) was significantly higher than that outside (mean ± SD: 0.26% ± 0.33%), *P <*0.001. The proportion of CD8^+^ T_RM_ in TLS (mean ± SD: 0.78% ± 0.72%) was significantly higher than that outside (mean ± SD: 0.55% ± 0.67%), *P <*0.05.

Survival analysis showed that both CD3^+^ T_RM_ and CD8^+^ T_RM_ in TLS could predict longer survival (median DFS 17.3 months vs. 6.7 months, 17.5 months vs. 12.7 months, *P*<0.05; **Figure 3C**). Likewise, CD4^+^ T_RM_ within TLS tended to prolong DFS of patients although there was no significant difference between groups (median 15.2 months vs. 6.9 months, *P* =0.078, **Figure 3C**). However, TRM outside the TLS had no effect on prognosis.

### The Relationship Between Patients Score, CD3^+^CD103^+^ T_RM_ Within TLS, and Various Clinical Parameters

The above findings suggested that T_RM_ is mainly located within the TLS, and the T_RM_ within the TLS can affect prognosis. Therefore, we next focused our research on T_RM_ inside TLS. The relationships between patient score, CD3^+^ T_RM_ within TLS, and clinical features of patients including sex, age, TNM stage, micropapillary, EGFR mutation, and smoking are shown in **Table 3**. The results showed that patients with stage IIIA LUAD had more mature TLS than patients with IIIB LUAD (*P* =0.041). There were no significant associations between other clinical features and patient scores, as well as between CD3^+^ T_RM_ and TLS.

**Table 3:** The relationship between patients score, CD3^+^CD103^+^T_RM_ within TLS and various clinical parameters in patients with stage III LUAD (n=49).

Variable	All cases (n)	Patients Score			*P*value	CD3^+^CD103^+^T_RM_ within TLS		*P*value
		grade1,n (%)	grade2,n (%)	grade3,n (%)		Low,n (%)	High,n (%)	
Gender								
Male	24	1 (4%)	12 (50%)	11 (46%)	0.118	5 (21%)	19 (79%)	>0.999
Female	25	5 (20%)	14 (56%)	6 (24%)		5 (20%)	20 (80%)	
Age (years)								
<60	28	3 (11%)	16 (57%)	9 (32%)	0.797	6 (21%)	22 (79%)	>0.999
≥60	21	3 (14%)	10 (48%)	8 (38%)		4 (19%)	17 (81%)	
T stage								
T_1_	29	2 (7%)	18 (62%)	9 (31%)	0.221	7 (24%)	22 (76%)	0.496
T_2_+T_3_+T_4_	20	4 (20%)	8 (40%)	8 (40%)		3 (15%)	17 (85%)	
N stage								
N_1_+N_2_	43	5 (12%)	22 (51%)	16 (37%)	0.61	10 (23%)	33 (77%)	0.324
N_3_	6	1 (17%)	4 (66%)	1 (17%)		0	6 (100%)	
TNM stage								
IIIA	39	3 (8%)	22 (56%)	14 (36%)	0.041	9 (23%)	30 (77%)	0.663
IIIB	10	3 (30%)	4 (40%)	3 (30%)		1 (10%)	9 (90%)	
Micropapillary								
Positive	22	3 (14%)	11 (50%)	8 (36%)	0.925	4 (18%)	18 (82%)	>0.999
Negative	27	3 (11%)	15 (56%)	9 (33%)		6 (22%)	21 (78%)	
EGFR mutation								
Positive	15	1 (7%)	11 (73%)	3 (20%)	0.279	2 (13%)	13 (87%)	0.175
Negative	10	3 (30%)	5 (50%)	2 (20%)		4 (40%)	6 (60%)	
Smoking								
Never	26	3 (12%)	14 (54%)	9 (34%)	0.986	4 (15%)	22 (85%)	0.483
Smoking	23	3 (13%)	12 (52%)	8 (35%)		6 (26%)	17 (74%)	

### Univariate Analysis of Clinical and Immune Characteristics Affecting DFS

Moreover, we analyzed the clinical and immune characteristics that affected the DFS of patients in this study. The results identified some univariate factors that could affect the DFS of patients, including the TLS number, TLS density, patient score, CD20^+^ B cells in TLS, FDC in TLS, CD103^+^ T_RM_, and CD8^+^CD103^+^ T_RM_ in TLS, average CD3^+^ CD103^+^ T_RM_, average CD4^+^CD103^+^ T_RM_, and average CD8^+^CD103^+^ T_RM_ (**Table 4**).

**Table 4:** Univariate analysis of clinical and immune characteristics affecting DFS of patients in the study.

Variable	HR (95%CI)	*P* value
Gender (Female vs. Male)	0.830 (0.473,1.459)	0.496
Age (≥60 y vs. <60 y)	0.622 (0.354,1.091)	0.086
T stage (T2+T3+T4 vs. T1)	0.905 (0.515,1.593)	0.728
N stage (N3 vs. N1+N2)	1.011 (0.429,2.384)	0.979
TNM stage (IIIB vs. IIIA)	1.284 (0.585,2.819)	0.486
Micropapillary (Negative vs. Positive)	1.259 (0.719,2.205)	0.412
Smoking (Never vs. Smoking)	0.911 (0.520,1.597)	0.741
Numbers of TLS (≥26 vs. <26)	0.490 (0.280,0.857)	0.012
Density of TLS,/mm^2^ (≥0.074 vs. <0.074)	0.459 (0.239,0.844)	0.006
Grade scores (grade2 vs. grade1)	0.418 (0.123,1.421)	0.039
(grade3 vs. grade1)	0.226 (0.047,1,078)	<0.001
CD3^+^T cell in TLS (≥19.17% vs. <19.17%)	1.326 (0.475,2.316)	0.316
CD4^+^T cell in TLS (≥14.08% vs. <14.08%)	0.665 (0.366,1.208)	0.196
CD8^+^T cell in TLS (≥18.81% vs. <18.81%)	1.483 (0.748,2.942)	0.202
CD20^+^B cell in TLS (≥17.46% vs. <17.46%)	0.571 (0.325,1.001)	0.044
Bcl6^+^B cell in TLS (≥0.05% vs. <0.05%)	0.564 (0.315,1.009)	0.070
CD21^+^FDC in TLS (≥0.56% vs. <0.56%)	0.375 (0.312,1.067)	0.004
CD103^+^cell in TLS (≥0.77% vs. <0.77%)	0.360 (0.097,1.338)	0.012
CD3^+^CD103^+^T_RM_ in TLS (≥0.48% vs. <0.48%)	0.433 (0.171,1.103)	0.012
CD4^+^CD103^+^T_RM_ in TLS (≥0.18% vs. <0.18%)	0.421 (0.093,1.888)	0.078
CD8^+^CD103^+^T_RM_ in TLS (≥0.28% vs. <0.28%)	0.386 (0.137,1.084)	0.005
CD3^+^T cell outside TLS (≥4.43% vs. <4.43%)	1.749 (0.939,3.258)	0.110
CD4^+^T cell outside TLS (≥3.75% vs. <3.75%)	1.606 (0.888,2.904)	0.089
CD8^+^T cell outside TLS (≥18.47% vs. <18.47%)	0.552 (0.286,1.072)	0.126
CD20^+^B cell outside TLS (≥26.14% vs. <26.14%)	0.599 (0.293,1.225)	0.176
CD3^+^CD103^+^T_RM_ outside TLS (≥0.37% vs. <0.37%)	1.514 (0.865,2.651)	0.135
CD4^+^CD103^+^T_RM_ outside TLS (≥0.16% vs. <0.16%)	1.591 (0.865,2.926)	0.102
CD8^+^CD103^+^T_RM_ outside TLS (≥0.25% vs. <0.25%)	1.654 (0.944,2.899)	0.076
CD3^+^T cell (≥8.52% vs. <8.52%)	0.487 (0.180,1.317)	0.052
CD4^+^T cell (≥12.67% vs. <12.67%)	0.637 (0.348,1.165)	0.160
CD8^+^T cell (≥6.37% vs. <6.37%)	0.472 (0.148,1.510)	0.072
CD20^+^B cell (≥19.04% vs. <19.04%)	0.555 (0.299,1.029)	0.092
Average CD3^+^CD103^+^T_RM_ (≥0.54% vs. <0.54%)	0.384 (0.136,1.080)	0.004
Average CD4^+^CD103^+^T_RM_ (≥0.26% vs. <0.26%)	0.490 (0.210,1.145)	0.026
Average CD8^+^CD103^+^T_RM_ (≥0.48% vs. <0.48%)	0.549 (0.281,1.037)	0.037

HR, hazard ratio; CI, confidence interval.

### Patients With High Score Had More T_RM_ in TLS

**Figure 4:**
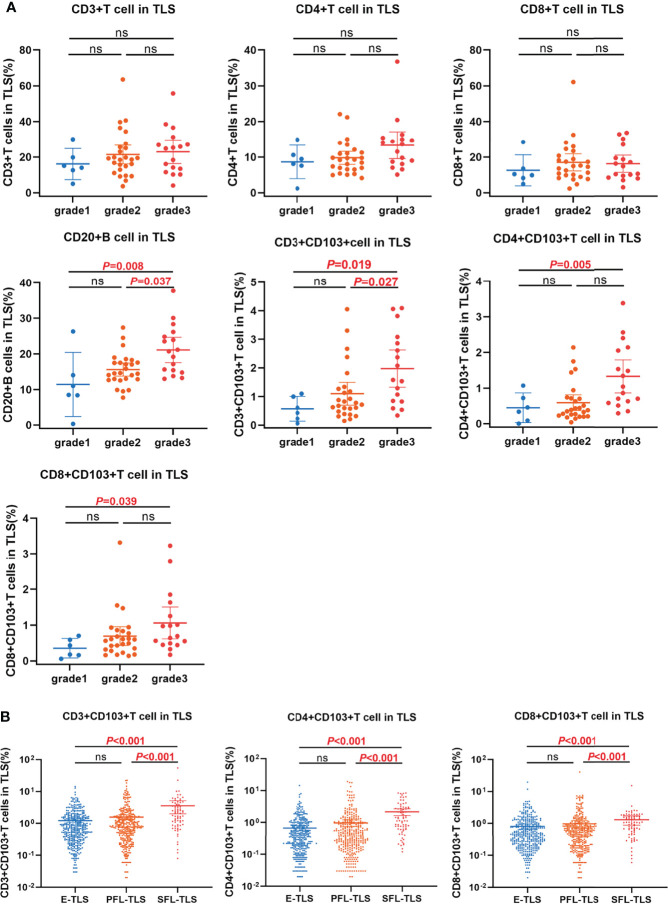
The relationship between T_RM_ distribution and TLSs maturation. **(A)** The distributions of immune subsets within TLS in patients with different TLS scores. The proportion of CD20^+^ B cell and CD3^+^CD103^+^ T_RM_ within TLS in grade 3 patients was significantly higher than that in grade 2 and grade 1 respectively. The proportion of CD4^+^CD103^+^ T_RM_ and CD8^+^CD103^+^ T_RM_ within TLS in grade 3 patients was significantly higher than that in grade 1. **(B)** The distribution of T_RM_ in E-TLS, PFL-TLS, and SFL-TLS. The proportions of CD3^+^CD103^+^ T_RM_, CD4^+^CD103^+^ T_RM_, and CD8^+^CD103^+^ T_RM_ within SFL-TLS were significantly higher than those in E-TLS and PFL-TLS, respectively. ns, non-significant.

We analyzed the distribution of immune subsets in the TLS in different grades of patients. The results showed that the proportion of CD20^+^ B cells in grade 3 patients was higher than that in grade 2 and grade 1 patients (mean ± SD: 21.08% ± 6.72% vs. 15.59% ± 4.47%, 21.08% ± 6.72% vs. 11.41% ± 7.84%, *P* =0.037, and *P* =0.008, respectively; **Figure 4A**). The proportion of CD3^+^ T_RM_ in grade 3 patients was higher than that in grade 2 and grade 1 patients (mean ± SD: 1.98% ± 1.23% vs. 1.10% ± 0.96%, 1.98% ± 1.23% vs. 0.57% ± 0.38%, *P* =0.027, and *P* =0.019, respectively; **Figure 4A**). Patients in grade 3 had higher CD4^+^ T_RM_ than patients in grade 1 (mean ± SD: 1.33% ± 0.87% vs. 0.59% ± 0.53%, *P* =0.005; **Figure 4A**). The proportion of CD8^+^ T_RM_ in grade 3 patients was higher than that in grade 1 (mean ± SD: 1.06% ± 0.84% vs. 0.69% ± 0.63%, *P* =0.039, **Figure 4A**).

Next, we analyzed the proportion of T_RM_ in all 807 TLSs, including E-TLSs, PFL-TLSs, and SFL-TLSs. The proportion of CD3^+^ T_RM_ in SFL-TLS was significantly higher than that of E-TLS and PFL-TLS (mean ± SD: 3.60% ± 6.80% vs. 1.57% ± 2.71%, 3.60% ± 6.80% vs. 1.23% ± 1.62%, *P*<0.001; **Figure 4B**), respectively. The proportion of CD4^+^ T_RM_ in SFL-TLS was significantly higher than that in E-TLS and PFL-TLS (mean ± SD: 2.17% ± 2.19% vs. 0.98% ± 2.21%, 2.17% ± 2.19% vs. 0.67% ± 1.19%, *P*<0.001; **Figure 4B**), respectively. The proportion of CD8^+^ T_RM_ in SFL-TLS was significantly higher than that in E-TLS and PFL-TLS (mean ± SD: 1.30% ± 1.78% vs. 0.98% ± 2.46%, 1.30% ± 1.78% vs. 0.81% ± 1.41%, *P*<0.001; **Figure 4B**), respectively.

### Patients With a Combination of T_RM_^High^ and Grade 3 Predicted a Better Prognosis

**Figure 5:**
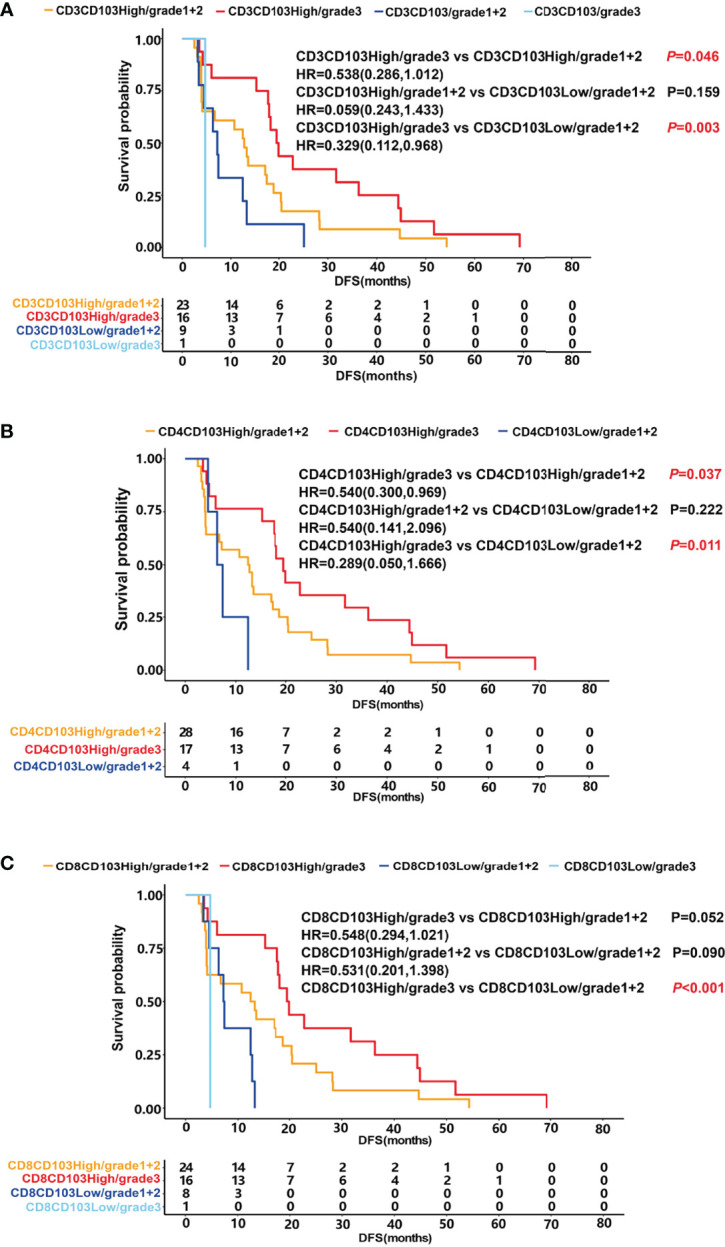
The relationship between T_RM_, patient score and prognosis. DFS was shown with Kaplan–Meier plots according to the combination of T_RM_ and patient score. **(A)** DFS of patients in the group of CD3CD103^High^ and grade 3 (median 19.7 months) was significantly higher than that of CD3CD103^High^ and grade 1 + 2 (median 12.7 months) and that of CD3CD103^Low^ and grade 1 + 2 (median 7.2 months), respectively, *P*<0.05. **(B)** DFS of patients in the group of CD4CD103^High^ and grade 3 (median 19.5 months) was significantly higher than that of CD4CD103^High^ and grade 1 + 2 (median 12.6 months) and that of CD4CD103^Low^ and grade 1 + 2 (median 6.9 months), respectively, *P*<0.05.  **(C)**. DFS of patients in the group of CD8CD103^High^ and grade 3 tended to be better than that of CD8CD103^High^ and grade 1 + 2 (median 19.7 months vs. 12.8 months, *P*=0.052), and significantly higher than that of CD8 CD103^Low^ and grade 1 + 2 (median DFS 19.7 months vs. 7.3 months, *P < *0.001).

Patients were stratified into four groups according to the proportion of CD103^+^ T_RM_ and patient scores. The prognosis of patients in the group of CD3CD103^High^ and grade 3 was significantly higher than that of CD3CD103^High^ and grade 1 + 2 (median DFS 19.7 months vs. 12.7 months, *P* =0.046) and that of CD3CD103^Low^ and grade 1 + 2 (median DFS 19.7 months vs. 7.2 months, *P* =0.003), respectively (**Figure 5A**). Patients in the CD4CD103^High^ and grade 3 groups had a significantly better prognosis than those in CD4CD103^High^ and grade 1 + 2 (median DFS 19.5 months vs. 12.6 months, *P* =0.037) and in CD4CD103^Low^ and grade 1 + 2 (median DFS 19.5 months vs. 6.9 months, *P* =0.011), respectively (**Figure 5B**). Similarly, the prognosis of patients in the group of CD8CD103^High^ and grade 3 tended to be better than that of CD8CD103^High^ and grade 1 + 2 (median DFS 19.7 months vs. 12.8 months, *P* =0.052), and significantly higher than that of CD8CD103^Low^ and grade 1 + 2 (median DFS 19.7 months vs. 7.3 months, *P <*0.001; **Figure 5C**). However, there was no significant difference in prognosis between patients in the CD103^High^ and grade 1 + 2 groups and the CD103^Low^ and grade 1 + 2 groups, regardless of the CD3^+^ T_RM_, CD4^+^ T_RM_, or CD8^+^ T_RM_ subsets.

## Discussion

In the present study, we evaluated the relationship among TLS maturity, clinical characteristics, and prognosis of patients with stage III LUAD. Although there is no standardized classification of TLS maturity, we used the classification method of TLS that Winder (26) had reported in colorectal carcinoma, and classified the TLSs into three mature stages, including E-TLSs, PFL-TLSs, and SFL-TLSs. For the first time, we divided patients into three levels based on the mature state of TLSs: 1) grade 1: only E-TLSs with no PFL-TLSs and SFL-TLSs; 2) grade 2: E-TLSs and PFL-TLSs in the tumor, and without SFL-TLS; and 3) grade 3: possess at least one SFL-TLS in the tumor tissue. The results showed that patients in grade 3 had the best DFS, followed by grade 2. The DFS of patients in grade 1 was the worst. This was consistent with the findings in colorectal cancer and lung squamous cell carcinoma that found that patients with GC reaction had a better prognosis (26, 27). This indicates that B cell maturity and humoral immunity play pivotal roles in the anti-tumor immune response.

In addition, we evaluated the distribution of CD4^+^ T_RM_ cells and CD8^+^ T_RM_ cells in the tumor tissue and found that the proportion of T_RM_ within TLSs was significantly higher than that outside, especially CD4^+^ T_RM_. The proportion of T_RM_ within TLSs was positively correlated with the prognosis of patients, while there was no significant association between the proportion of T_RM_ outside TLSs and prognosis. Furthermore, we compared the proportions of different immune subsets in LUAD patients of different grades. The proportions of CD20^+^ B cells and CD3^+^ T_RM_ in grade 3 patients were significantly higher than those in grade 1 and grade 2, respectively. The proportions of CD4^+^CD103^+^ T_RM_ and CD8^+^CD103^+^ T_RM_ were significantly higher in grade 3 patients than in grade 1 patients (*P*<0.05). We then analyzed the proportion difference of T_RM_ in different maturities of TLSs. The proportions of CD3^+^ T_RM_, CD4^+^ T_RM_, and CD8^+^ T_RM_ in SFL-TLSs were significantly higher than those in E-TLSs and PFL-TLSs, respectively (*P*<0.05). All these results indicate that there is a close relationship between T_RM_ and TLS maturity.

In the subsequent prognosis analysis, the data showed that patients with both more mature TLSs and a higher proportion of CD103^+^ T_RM_ had a much better prognosis. CD3^+^ T_RM_, CD4^+^ T_RM_, and CD8^+^ T_RM_ showed similar results. The data further confirmed that CD103^+^ T_RM_ was closely related to the maturation of TLSs.

Although the exact mechanism by which T_RM_ preferentially located into TLS had not been clarified, it was reported that CXCL13 was the key molecular determinant of TLS formation in the TME (27–30). Activated CD103^+^CTLs were involved in the migration of B cells to tumor *via* production of CXCL13. The high mutation load and CD8^+^ T cell–rich tumors showed higher expression of CXCL13 and ITGAE (CD103) and that they presented with significantly higher numbers of B cells in a variety of tumors (30). A previous study on the distribution of CD8^+^CD103^+^ T_RM_ in gastric carcinoma reported similar results. CD103^+^ T cells were located around TLSs, and patients with CD103^High^ had more TLSs (24). Furthermore, patients who were CD103^high^ and TLS^rich^ had a better prognosis than other groups (24). However, this study mainly focused on CD8^+^ subsets and there was no analysis of the relationship between TLS maturity and CD103^+^ T_RM_. Another study identified a new subset of CD4^+^ Th-CXCL13 with tumor-resident gene characteristics in NPC (31). CD4^+^ Th-CXCL13 recruits tumor-associated B cells and induces plasma cell differentiation and immunoglobulin production through interleukin-21 (IL-21) secretion and CD84 interactions in TLSs. In a mouse model of influenza viral infection, Young Min Son et al. reported a population of lung-resident helper CD4^+^ T cells (CD4^+^ T_RH_) that developed after viral clearance. They found that the formation of CD4^+^ T_RH_ is dependent on transcription factors involved in the feather of follicular T cells and resident T cells, including BCL6 and Bhlhe40. CD4^+^ T_RH_ could promote the development of protective B cells and CD8^+^ T cell responses through IL-21 dependent mechanism (32). Moreover, B cells in TLSs can function as antigen-presenting cells; they highly express the co-stimulatory molecules CD86 and CD80 and facilitate tumor antigen-specific T-cell responses, including CD8^+^ TIL and CD4^+^ TIL responses (33). Bradley et al. have demonstrated that B cells play important roles in memory CD4^+^ T cell generation and differentiation because mice in a B cell knockout model did not develop memory CD4^+^ T cells (34). These results indicated that there might be synergy function between T_RM_ and TLSs in the antitumor response.

In conclusion, our data highlight the proportion of T_RM_ within TLSs was significantly increased with the maturation of TLSs. When we divided patients into three levels including grade 1, grade 2 and grade 3 according to the presence of different maturity of TLSs, the proportions of CD4^+^CD103^+^T_RM_ and CD8^+^CD103^+^T_RM_ in grade 3 of patients were significantly higher than grade 1 and grade 2. These results indicate a close relationship between CD103^+^T_RM_ and TLS maturity. Furthermore, patients with a combination feature of grade 3 and CD103^+^ ${\text{T}}_{\text{RM}}^{\text{High}}$ exhibited a good prognosis. The combination of TLS maturity and CD103^+^ T_RM_ proportion could be used as a biomarker to predict the prognosis of LUAD patients.

## Data Availability Statement

The original contributions presented in the study are included in the article/**Supplementary Material**. Further inquiries can be directed to the corresponding authors.

## Ethics Statement

The studies involving human participants were reviewed and approved by Ethics Committee of the Tianjin Medical University Cancer Institute and Hospital. The patients/participants provided their written informed consent to participate in this study.

## Author Contributions

HZ and XR designed the experiments. HW performed the experiments. HZ and HW performed the analyses and wrote the manuscript. HW and YZ collected clinical data. QS and XR revised the manuscript. All authors have commented on and approved the manuscript. HZ and HW contributed equally to this work. All authors contributed to the article and approved the submitted version.

## Funding

The work in this study was supported by a grant from the National Natural Science Foundation of China (Grant No. U20A20375).

## Conflict of Interest

The authors declare that the research was conducted in the absence of any commercial or financial relationships that could be construed as a potential conflict of interest.

## Publisher’s Note

All claims expressed in this article are solely those of the authors and do not necessarily represent those of their affiliated organizations, or those of the publisher, the editors and the reviewers. Any product that may be evaluated in this article, or claim that may be made by its manufacturer, is not guaranteed or endorsed by the publisher.

## References

[1] Hsu ML Naidoo J. Principles of Immunotherapy in non-Small Cell Lung Cancer. Thorac Surg Clin (2020) 30(2):187–98. doi: 10.1016/j.thorsurg.2020.01.009 32327177

[2] Suresh K Naidoo J Lin CT Danoff S. Immune Checkpoint Immunotherapy for non-Small Cell Lung Cancer: Benefits and Pulmonary Toxicities. Chest (2018) 154(6):1416–23. doi: 10.1016/j.chest.2018.08.1048 PMC6335259 30189190

[3] Tumeh PC Harview CL Yearley JH Shintaku IP Taylor EJ Robert L. PD-1 Blockade Induces Responses by Inhibiting Adaptive Immune Resistance. Nature (2014) 515(7528):568–71. doi: 10.1038/nature13954 PMC4246418 25428505

[4] Helmink BA Reddy SM Gao J Zhang S Basar R Thakur R. B Cells and Tertiary Lymphoid Structures Promote Immunotherapy Response. Nature (2020) 577(7791):549–55. doi: 10.1038/s41586-019-1922-8 PMC8762581 31942075

[5] Cabrita R Lauss M Sanna A Donia M Skaarup Larsen M Mitra S. Tertiary Lymphoid Structures Improve Immunotherapy and Survival in Melanoma. Nature (2020) 577(7791):561–5. doi: 10.1038/s41586-019-1914-8 31942071

[6] Petitprez F de Reyniès A Keung EZ Chen TW Sun CM Calderaro J. B Cells are Associated With Survival and Immunotherapy Response in Sarcoma. Nature (2020) 577(7791):556–60. doi: 10.1038/s41586-019-1906-8 31942077

[7] Sautès-Fridman C Petitprez F Calderaro J Fridman WH. Tertiary Lymphoid Structures in the Era of Cancer Immunotherapy. Nat Rev Cancer (2019) 19(6):307–25. doi: 10.1038/s41568-019-0144-6 31092904

[8] Zhao H Wang H Zhou Q Ren X. Insights Into Tertiary Lymphoid Structures in the Solid Tumor Microenvironment: Anti-Tumor Mechanism, Functional Regulation, and Immunotherapeutic Strategies. Cancer Biol Med (2021) 18(4):981–91. doi: 10.20892/j.issn.2095-3941.2021.0029 PMC8610165 34553849

[9] Dieu-Nosjean MC Antoine M Danel C Heudes D Wislez M Poulot V. Long-Term Survival for Patients With non-Small-Cell Lung Cancer With Intratumoral Lymphoid Structures. J Clin Oncol (2008) 26(27):4410–7. doi: 10.1200/JCO.2007.15.0284 18802153

[10] Kroeger DR Milne K Nelson BH. Tumor-Infiltrating Plasma Cells are Associated With Tertiary Lymphoid Structures, Cytolytic T-Cell Responses, and Superior Prognosis in Ovarian Cancer. Clin Cancer Res (2016) 22(12):3005–15. doi: 10.1158/1078-0432.CCR-15-2762 26763251

[11] Rodriguez AB Engelhard VH. Insights Into Tumor-Associated Tertiary Lymphoid Structures: Novel Targets for Antitumor Immunity and Cancer Immunotherapy. Cancer Immunol Res (2020) 8(11):1338–45. doi: 10.1158/2326-6066.CIR-20-0432 PMC7643396 33139300

[12] Munoz-Erazo L Rhodes JL Marion VC Kemp RA. Tertiary Lymphoid Structures in Cancer – Considerations for Patient Prognosis. Cell Mol Immunol (2020) 17(6):570–5. doi: 10.1038/s41423-020-0457-0 PMC7264315 32415259

[13] Yamakoshi Y Tanaka H Sakimura C Deguchi S Mori T Tamura T. Immunological Potential of Tertiary Lymphoid Structures Surrounding the Primary Tumor in Gastric Cancer. Int J Oncol (2020) 57(1):171–82. doi: 10.3892/ijo.2020.5042 PMC7252463 32319601

[14] Martinet L Garrido I Filleron T Le Guellec SL Bellard E Fournie JJ. Human Solid Tumors Contain High Endothelial Venules: Association With T- and B-Lymphocyte Infiltration and Favorable Prognosis in Breast Cancer. Cancer Res (2011) 71(17):5678–87. doi: 10.1158/0008-5472.CAN-11-0431 21846823

[15] Djenidi F Adam J Goubar A Durgeau A Meurice G de Montpréville V. CD8+CD103+ Tumor-Infiltrating Lymphocytes are Tumor-Specific Tissue-Resident Memory T Cells and a Prognostic Factor for Survival in Lung Cancer Patients. J Immunol (2015) 194(7):3475–86. doi: 10.4049/jimmunol.1402711 25725111

[16] Ganesan AP Clarke J Wood O Garrido-Martin EM Chee SJ Mellows T. Tissue-Resident Memory Features are Linked to the Magnitude of Cytotoxic T Cell Responses in Human Lung Cancer. Nat Immunol (2017) 18(8):940–50. doi: 10.1038/ni.3775 PMC6036910 28628092

[17] Schenkel JM Masopust D. Tissue-Resident Memory T Cells. Immunity (2014) 41(6):886–97. doi: 10.1016/j.immuni.2014.12.007 PMC4276131 25526304

[18] Park SL Gebhardt T Mackay LK. Tissue-Resident Memory T Cells in Cancer Immunosurveillance. Trends Immunol (2019) 40(8):735–47. doi: 10.1016/j.it.2019.06.002 31255505

[19] Oja AE Piet B van der Zwan D Blaauwgeers H Mensink M de Kivit S. Functional Heterogeneity of CD4+ Tumor-Infiltrating Lymphocytes With a Resident Memory Phenotype in NSCLC. Front Immunol (2018) 9:2654. doi: 10.3389/fimmu.2018.02654 30505306 PMC6250821

[20] Gaudreau PO Negrao MV Mitchell KG Reuben A Corsini EM Li J. Neoadjuvant Chemotherapy Increases Cytotoxic T Cell, Tissue Resident Memory T Cell, and B Cell Infiltration in Resectable NSCLC. J Thorac Oncol (2021) 16(1):127–39. doi: 10.1016/j.jtho.2020.09.027 PMC7775914 33096269

[21] Ida S Takahashi H Kawabata-Iwakawa R Mito I Tada H Chikamatsu K. Tissue-Resident Memory T Cells Correlate With the Inflammatory Tumor Microenvironment and Improved Prognosis in Head and Neck Squamous Cell Carcinoma. Oral Oncol (2021) 122:105508. doi: 10.1016/j.oraloncology.2021.105508 34507204

[22] Savas P Virassamy B Ye C Salim A Mintoff CP Caramia F. Single-Cell Profiling of Breast Cancer T Cells Reveals a Tissue-Resident Memory Subset Associated With Improved Prognosis. Nat Med (2018) 24(7):986–93. doi: 10.1038/s41591-018-0078-7 29942092

[23] Banchereau R Chitre AS Scherl A Wu TD Patil NS de Almeida P. Intratumoral CD103+ CD8+ T Cells Predict Response to PD-L1 Blockade. J Immunother Cancer (2021) 9(4):e002231. doi: 10.1136/jitc-2020-002231 33827905 PMC8032254

[24] Mori T Tanaka H Suzuki S Deguchi S Yamakoshi Y Yoshii M. Tertiary Lymphoid Structures Show Infiltration of Effective Tumor-Resident T Cells in Gastric Cancer. Cancer Sci (2021) 112(5):1746–57. doi: 10.1111/cas.14888 PMC8088970 33735485

[25] Yamaguchi K Ito M Ohmura H Hanamura F Nakano M Tsuchihashi K. Helper T Cell-Dominant Tertiary Lymphoid Structures are Associated With Disease Relapse of Advanced Colorectal Cancer. Oncoimmunology (2020) 9(1):1724763. doi: 10.1080/2162402X.2020.1724763 32117589 PMC7028340

[26] Posch F Silina K Leibl S Mündlein A Moch H Siebenhüner A. Maturation of Tertiary Lymphoid Structures and Recurrence of Stage II and III Colorectal Cancer. Oncoimmunology (2018) 7(2):e1378844. doi: 10.1080/2162402X.2017.1378844 29416939 PMC5798199

[27] Siliņa K Soltermann A Attar FM Casanova R Uckeley ZM Thut H. Germinal Centers Determine the Prognostic Relevance of Tertiary Lymphoid Structures and are Impaired by Corticosteroids in Lung Squamous Cell Carcinoma. Cancer Res (2018) 78(5):1308–20. doi: 10.1158/0008-5472.CAN-17-1987 29279354

[28] Gräbner R Lötzer K Döpping S Hildner M Radke D Beer M. Lymphotoxin Beta Receptor Signaling Promotes Tertiary Lymphoid Organogenesis in the Aorta Adventitia of Aged ApoE-/- Mice. J Exp Med (2009) 206(1):233–48. doi: 10.1084/jem.20080752 PMC2626665 19139167

[29] Fleige H Ravens S Moschovakis GL Bölter J Willenzon S Sutter G. IL-17-Induced CXCL12 Recruits B Cells and Induces Follicle Formation in BALT in the Absence of Differentiated FDCs. J Exp Med (2014) 211(4):643–51. doi: 10.1084/jem.20131737 PMC3978277 24663215

[30] Workel HH Lubbers JM Arnold R Prins TM van der Vlies P de Lange K. A Transcriptionally Distinct Cxcl13+Cd103+Cd8+ T-Cell Population Is Associated With B-Cell Recruitment and Neoantigen Load in Human Cancer. Cancer Immunol Res (2019) 7(5):784–96. doi: 10.1158/2326-6066.CIR-18-0517 30872264

[31] Li JP Wu CY Chen MY Liu SX Yan SM Kang YF. PD-1+CXCR5-CD4+ Th-CXCL13 Cell Subset Drives B Cells Into Tertiary Lymphoid Structures of Nasopharyngeal Carcinoma. J Immunother Cancer (2021) 9(7):e002101. doi: 10.1136/jitc-2020-002101 34253636 PMC8276302

[32] Son YM Cheon IS Wu Y Li C Wang Z Gao X. Tissue-Resident CD4+ T Helper Cells Assist the Development of Protective Respiratory B and CD8+ T Cell Memory Responses. Sci Immunol (2021) 6(55):eabb6852. doi: 10.1126/sciimmunol.abb6852 33419791 PMC8056937

[33] Behr DS Peitsch WK Hametner C Lasitschka F Houben R Schönhaar K. Prognostic Value of Immune Cell Infiltration, Tertiary Lymphoid Structures and PD-L1 Expression in Merkel Cell Carcinomas. Int J Clin Exp Pathol (2014) 7(11):7610–21. PMC4270630 25550797

[34] Linton PJ Harbertson J Bradley LM. A Critical Role for B Cells in the Development of Memory CD4 Cells. J Immunol (2000) 165(10):5558–65. doi: 10.4049/jimmunol.165.10.5558 11067910

