# Supplementary material for: Tumor-Resident T Cells, Associated With Tertiary Lymphoid Structure Maturity, Improve Survival in Patients With Stage III Lung Adenocarcinoma
Source: Front Immunol. 2022 May 19;13:877689. doi: 10.3389/fimmu.2022.877689 (PMC9161276; doi:10.3389/fimmu.2022.877689)
Supplement: Supplementary file 3 [file Table_3.docx]

**Supplementary Table 3.** Image information for each patient(n=49)

| **Patients** | **CD20+ aggregations** | **TLS**  **scan areas** | **E-TLS** | **PFL-TLS** | **SFL-TLS** | **Area**  **outside TLS** |
| --- | --- | --- | --- | --- | --- | --- |
| 452794 | 18 | 18 | 5 | 12 | 1 | 3 |
| 454709 | 14 | 14 | 5 | 8 | 1 | 3 |
| 454122 | 13 | 13 | 8 | 5 | 0 | 3 |
| 454352 | 25 | 25 | 13 | 12 | 0 | 3 |
| 454443 | 81 | 36 | 19 | 17 | 0 | 3 |
| 454654 | 37 | 37 | 18 | 11 | 8 | 3 |
| 454726 | 11 | 11 | 6 | 5 | 0 | 3 |
| 455021 | 4 | 4 | 2 | 1 | 1 | 3 |
| 455596 | 7 | 7 | 3 | 4 | 0 | 3 |
| 455661 | 2 | 2 | 2 | 0 | 0 | 3 |
| 456119 | 9 | 9 | 4 | 4 | 1 | 3 |
| 456430 | 7 | 7 | 5 | 2 | 0 | 3 |
| 456740 | 15 | 15 | 9 | 6 | 0 | 3 |
| 456832 | 1 | 1 | 1 | 0 | 0 | 3 |
| 457178 | 29 | 29 | 15 | 14 | 0 | 3 |
| 457202 | 19 | 19 | 5 | 12 | 2 | 3 |
| 458054 | 9 | 9 | 4 | 5 | 0 | 3 |
| 458204 | 17 | 17 | 8 | 9 | 0 | 3 |
| 458400 | 2 | 2 | 2 | 0 | 0 | 4 |
| 461580 | 7 | 7 | 3 | 4 | 0 | 3 |
| 463143 | 3 | 3 | 2 | 1 | 0 | 3 |
| 466285 | 8 | 8 | 2 | 3 | 3 | 3 |
| 467250 | 53 | 45 | 33 | 12 | 0 | 3 |
| 468166 | 21 | 21 | 12 | 9 | 0 | 3 |
| 468631 | 71 | 26 | 6 | 16 | 4 | 3 |
| 469392 | 1 | 1 | 1 | 0 | 0 | 3 |
| 470211 | 19 | 19 | 11 | 8 | 0 | 3 |
| 471052 | 27 | 27 | 13 | 9 | 5 | 3 |
| 471425 | 17 | 17 | 7 | 7 | 3 | 3 |
| 471479 | 33 | 33 | 14 | 17 | 2 | 3 |
| 471955 | 8 | 8 | 3 | 5 | 0 | 3 |
| 472594 | 21 | 21 | 13 | 8 | 0 | 3 |
| 472974 | 4 | 4 | 1 | 3 | 0 | 3 |
| 473001 | 7 | 7 | 4 | 3 | 0 | 3 |
| 473600 | 18 | 18 | 10 | 8 | 0 | 3 |
| 473601 | 48 | 48 | 12 | 25 | 11 | 4 |
| 474085 | 12 | 12 | 6 | 6 | 0 | 3 |
| 474223 | 19 | 19 | 15 | 4 | 0 | 3 |
| 474312 | 19 | 19 | 8 | 11 | 0 | 3 |
| 474313 | 13 | 13 | 6 | 7 | 0 | 3 |
| 475474 | 13 | 13 | 3 | 10 | 0 | 3 |
| 475944 | 4 | 4 | 1 | 2 | 1 | 3 |
| 476531 | 4 | 4 | 4 | 0 | 0 | 3 |
| 476709 | 31 | 31 | 8 | 5 | 18 | 3 |
| 476760 | 14 | 14 | 3 | 11 | 0 | 3 |
| 476932 | 45 | 34 | 9 | 15 | 10 | 3 |
| 477265 | 29 | 29 | 14 | 14 | 1 | 5 |
| 477301 | 1 | 1 | 1 | 0 | 0 | 3 |
| 478011 | 26 | 26 | 18 | 7 | 1 | 3 |
| **Total** | 916 | 807 | 377 | 357 | 73 | 151 |
