# Supplementary material for: Tumor-Resident T Cells, Associated With Tertiary Lymphoid Structure Maturity, Improve Survival in Patients With Stage III Lung Adenocarcinoma
Source: Front Immunol. 2022 May 19;13:877689. doi: 10.3389/fimmu.2022.877689 (PMC9161276; doi:10.3389/fimmu.2022.877689)
Supplement: Supplementary file 4 [file Table_4.docx]

**1. R Figure 2A left:**

#install.packages("survival")

#install.packages("survminer")

rm(list=ls())

library(survival)

library(survminer)

inputFile="input.txt"

outFile="survival.pdf"

var="TLS"

setwd("C:/R语言 survivalCutoff")

rt=read.table(inputFile, header=T, sep="\t", check.names=F)

rt=rt[,c("futime","fustat",var)]

rt$futime=rt$futime/30 #月就除以30年除以365

colnames(rt)=c("futime","fustat","var")

res.cut=surv_cutpoint(rt, time = "futime", event = "fustat",variables =c("var"))

res.cut

res.cat=surv_categorize(res.cut)

fit=survfit(Surv(futime, fustat) ~var, data = res.cat)

diff=survdiff(Surv(futime, fustat) ~var,data =res.cat)

pValue=1-pchisq(diff$chisq,df=1)

if(pValue<0.001){

pValue="p<0.001"

}else{

pValue=paste0("p=",sprintf("%.03f",pValue))

}

surPlot=ggsurvplot(fit,

data=res.cat,

conf.int=TRUE,

pval=pValue,

pval.size=5,

legend.labs=c("High", "Low"), #分组名称，可换

legend.title=var,

xlab="Time(months)", #X轴名称，可以换

break.time.by = 10, #X轴间距，可换

risk.table.title="",

palette=c("red", "blue"),

risk.table=T,

risk.table.height=.25)

pdf(file=outFile,onefile = FALSE,width = 5,height =5)

print(surPlot)

dev.off()

**2. R Figure 2A right:**

#install.packages("survival")

#install.packages("survminer")

rm(list=ls())

library(survival)

library(survminer)

inputFile="input.txt"

outFile="survival.pdf"

var="TLS/mm2"

setwd("C:/R语言 survivalCutoff")

rt=read.table(inputFile, header=T, sep="\t", check.names=F)

rt=rt[,c("futime","fustat",var)]

rt$futime=rt$futime/30 #月就除以30年除以365

colnames(rt)=c("futime","fustat","var")

res.cut=surv_cutpoint(rt, time = "futime", event = "fustat",variables =c("var"))

res.cut

res.cat=surv_categorize(res.cut)

fit=survfit(Surv(futime, fustat) ~var, data = res.cat)

diff=survdiff(Surv(futime, fustat) ~var,data =res.cat)

pValue=1-pchisq(diff$chisq,df=1)

if(pValue<0.001){

pValue="p<0.001"

}else{

pValue=paste0("p=",sprintf("%.03f",pValue))

}

surPlot=ggsurvplot(fit,

data=res.cat,

conf.int=TRUE,

pval=pValue,

pval.size=5,

legend.labs=c("High", "Low"), #分组名称，可换

legend.title=var,

xlab="Time(months)", #X轴名称，可以换

break.time.by = 10, #X轴间距，可换

risk.table.title="",

palette=c("red", "blue"),

risk.table=T,

risk.table.height=.25)

pdf(file=outFile,onefile = FALSE,width = 5,height =5)

print(surPlot)

dev.off()

**3. R Figure 2B:**

rm(list = ls())

options(stringsAsFactors = F)

phe=read.csv(file="phe.csv", header=T)

colnames(phe)

phe$futime_month=round(phe$futime/30,1)

library(survival)

library(survminer)

sfit <- survfit(Surv(phe$futime_month, phe$fustat)~phe$GRADE, data=phe)

summary(sfit)

gg=ggsurvplot(sfit,

palette = c("blue","orange" ,"red"),

risk.table =TRUE,

conf.int =F,

legend.title = "",

xlab ="Time(months)",

break.x.by = 10,

risk.table.title=""

)

gg

**4. R Figure3C CD3CD103T cell in TLS:**

#install.packages("survival")

#install.packages("survminer")

rm(list=ls())

library(survival)

library(survminer)

inputFile="input.txt"

outFile="survival.pdf"

var="CD3CD103"

setwd("C:/R语言 survivalCutoff")

rt=read.table(inputFile, header=T, sep="\t", check.names=F)

rt=rt[,c("futime","fustat",var)]

rt$futime=rt$futime/30 #月就除以30年除以365

colnames(rt)=c("futime","fustat","var")

res.cut=surv_cutpoint(rt, time = "futime", event = "fustat",variables =c("var"))

res.cut

res.cat=surv_categorize(res.cut)

fit=survfit(Surv(futime, fustat) ~var, data = res.cat)

diff=survdiff(Surv(futime, fustat) ~var,data =res.cat)

pValue=1-pchisq(diff$chisq,df=1)

if(pValue<0.001){

pValue="p<0.001"

}else{

pValue=paste0("p=",sprintf("%.03f",pValue))

}

surPlot=ggsurvplot(fit,

data=res.cat,

conf.int=TRUE,

pval=pValue,

pval.size=5,

legend.labs=c("High", "Low"), #分组名称，可换

legend.title=var,

xlab="Time(months)", #X轴名称，可以换

break.time.by = 10, #X轴间距，可换

risk.table.title="",

palette=c("red", "blue"),

risk.table=T,

risk.table.height=.25)

pdf(file=outFile,onefile = FALSE,width = 5,height =5)

print(surPlot)

dev.off()

**5. R Figure3C CD3CD103T cell outside TLS:**

#install.packages("survival")

#install.packages("survminer")

rm(list=ls())

library(survival)

library(survminer)

inputFile="input.txt"

outFile="survival.pdf"

var="CD3CD103"

setwd("C:/R语言 survivalCutoff")

rt=read.table(inputFile, header=T, sep="\t", check.names=F)

rt=rt[,c("futime","fustat",var)]

rt$futime=rt$futime/30 #月就除以30年除以365

colnames(rt)=c("futime","fustat","var")

res.cut=surv_cutpoint(rt, time = "futime", event = "fustat",variables =c("var"))

res.cut

res.cat=surv_categorize(res.cut)

fit=survfit(Surv(futime, fustat) ~var, data = res.cat)

diff=survdiff(Surv(futime, fustat) ~var,data =res.cat)

pValue=1-pchisq(diff$chisq,df=1)

if(pValue<0.001){

pValue="p<0.001"

}else{

pValue=paste0("p=",sprintf("%.03f",pValue))

}

surPlot=ggsurvplot(fit,

data=res.cat,

conf.int=TRUE,

pval=pValue,

pval.size=5,

legend.labs=c("High", "Low"), #分组名称，可换

legend.title=var,

xlab="Time(months)", #X轴名称，可以换

break.time.by = 10, #X轴间距，可换

risk.table.title="",

palette=c("red", "blue"),

risk.table=T,

risk.table.height=.25)

pdf(file=outFile,onefile = FALSE,width = 5,height =5)

print(surPlot)

dev.off()

**6. R Figure3C CD4CD103T cell in TLS:**

#install.packages("survival")

#install.packages("survminer")

rm(list=ls())

library(survival)

library(survminer)

inputFile="input.txt"

outFile="survival.pdf"

var="CD4CD103"

setwd("C:/R语言 survivalCutoff")

rt=read.table(inputFile, header=T, sep="\t", check.names=F)

rt=rt[,c("futime","fustat",var)]

rt$futime=rt$futime/30 #月就除以30年除以365

colnames(rt)=c("futime","fustat","var")

res.cut=surv_cutpoint(rt, time = "futime", event = "fustat",variables =c("var"))

res.cut

res.cat=surv_categorize(res.cut)

fit=survfit(Surv(futime, fustat) ~var, data = res.cat)

diff=survdiff(Surv(futime, fustat) ~var,data =res.cat)

pValue=1-pchisq(diff$chisq,df=1)

if(pValue<0.001){

pValue="p<0.001"

}else{

pValue=paste0("p=",sprintf("%.03f",pValue))

}

surPlot=ggsurvplot(fit,

data=res.cat,

conf.int=TRUE,

pval=pValue,

pval.size=5,

legend.labs=c("High", "Low"), #分组名称，可换

legend.title=var,

xlab="Time(months)", #X轴名称，可以换

break.time.by = 10, #X轴间距，可换

risk.table.title="",

palette=c("red", "blue"),

risk.table=T,

risk.table.height=.25)

pdf(file=outFile,onefile = FALSE,width = 5,height =5)

print(surPlot)

dev.off()

**7. R Figure3C CD4CD103T cell outside TLS:**

#install.packages("survival")

#install.packages("survminer")

rm(list=ls())

library(survival)

library(survminer)

inputFile="input.txt"

outFile="survival.pdf"

var="CD4CD103"

setwd("C:/R语言 survivalCutoff")

rt=read.table(inputFile, header=T, sep="\t", check.names=F)

rt=rt[,c("futime","fustat",var)]

rt$futime=rt$futime/30 #月就除以30年除以365

colnames(rt)=c("futime","fustat","var")

res.cut=surv_cutpoint(rt, time = "futime", event = "fustat",variables =c("var"))

res.cut

res.cat=surv_categorize(res.cut)

fit=survfit(Surv(futime, fustat) ~var, data = res.cat)

diff=survdiff(Surv(futime, fustat) ~var,data =res.cat)

pValue=1-pchisq(diff$chisq,df=1)

if(pValue<0.001){

pValue="p<0.001"

}else{

pValue=paste0("p=",sprintf("%.03f",pValue))

}

surPlot=ggsurvplot(fit,

data=res.cat,

conf.int=TRUE,

pval=pValue,

pval.size=5,

legend.labs=c("High", "Low"), #分组名称，可换

legend.title=var,

xlab="Time(months)", #X轴名称，可以换

break.time.by = 10, #X轴间距，可换

risk.table.title="",

palette=c("red", "blue"),

risk.table=T,

risk.table.height=.25)

pdf(file=outFile,onefile = FALSE,width = 5,height =5)

print(surPlot)

dev.off()

**8. R Figure3C CD8CD103T cell in TLS:**

#install.packages("survival")

#install.packages("survminer")

rm(list=ls())

library(survival)

library(survminer)

inputFile="input.txt"

outFile="survival.pdf"

var="CD8CD103"

setwd("C:/R语言 survivalCutoff")

rt=read.table(inputFile, header=T, sep="\t", check.names=F)

rt=rt[,c("futime","fustat",var)]

rt$futime=rt$futime/30 #月就除以30年除以365

colnames(rt)=c("futime","fustat","var")

res.cut=surv_cutpoint(rt, time = "futime", event = "fustat",variables =c("var"))

res.cut

res.cat=surv_categorize(res.cut)

fit=survfit(Surv(futime, fustat) ~var, data = res.cat)

diff=survdiff(Surv(futime, fustat) ~var,data =res.cat)

pValue=1-pchisq(diff$chisq,df=1)

if(pValue<0.001){

pValue="p<0.001"

}else{

pValue=paste0("p=",sprintf("%.03f",pValue))

}

surPlot=ggsurvplot(fit,

data=res.cat,

conf.int=TRUE,

pval=pValue,

pval.size=5,

legend.labs=c("High", "Low"), #分组名称，可换

legend.title=var,

xlab="Time(months)", #X轴名称，可以换

break.time.by = 10, #X轴间距，可换

risk.table.title="",

palette=c("red", "blue"),

risk.table=T,

risk.table.height=.25)

pdf(file=outFile,onefile = FALSE,width = 5,height =5)

print(surPlot)

dev.off()

**9. R Figure3C CD8CD103T cell outside TLS:**

#install.packages("survival")

#install.packages("survminer")

rm(list=ls())

library(survival)

library(survminer)

inputFile="input.txt"

outFile="survival.pdf"

var="CD8CD103"

setwd("C:/R语言 survivalCutoff")

rt=read.table(inputFile, header=T, sep="\t", check.names=F)

rt=rt[,c("futime","fustat",var)]

rt$futime=rt$futime/30 #月就除以30年除以365

colnames(rt)=c("futime","fustat","var")

res.cut=surv_cutpoint(rt, time = "futime", event = "fustat",variables =c("var"))

res.cut

res.cat=surv_categorize(res.cut)

fit=survfit(Surv(futime, fustat) ~var, data = res.cat)

diff=survdiff(Surv(futime, fustat) ~var,data =res.cat)

pValue=1-pchisq(diff$chisq,df=1)

if(pValue<0.001){

pValue="p<0.001"

}else{

pValue=paste0("p=",sprintf("%.03f",pValue))

}

surPlot=ggsurvplot(fit,

data=res.cat,

conf.int=TRUE,

pval=pValue,

pval.size=5,

legend.labs=c("High", "Low"), #分组名称，可换

legend.title=var,

xlab="Time(months)", #X轴名称，可以换

break.time.by = 10, #X轴间距，可换

risk.table.title="",

palette=c("red", "blue"),

risk.table=T,

risk.table.height=.25)

pdf(file=outFile,onefile = FALSE,width = 5,height =5)

print(surPlot)

dev.off()

**10. R Figure5A:**

rm(list = ls())

options(stringsAsFactors = F)

phe=read.csv(file="phe_.csv", header=T)

colnames(phe)

phe$futime_month=round(phe$futime/30,1)

library(survival)

library(survminer)

sfit <- survfit(Surv(phe$futime_month, phe$fustat)~phe$group, data=phe)

summary(sfit)

gg=ggsurvplot(sfit,

palette = c("orange","red","blue","sky blue"),

risk.table =TRUE,

conf.int =F,

legend.title = "",

legend.labs = c("CD3CD103High/Grade3", "CD3CD103High/Grade1+2","CD3CD103Low/Grade1+2","CD3CD103Low/Grade3"),

xlab ="Time(months)",

break.x.by = 10,

risk.table.title="",

break.time.by = 10, #X轴间距，可换

risk.table.height=.25,

font.x = 14,

font.y =14,

font.legend=10,

risk.table.fontsize=6)

gg

**11. R Figure5B:**

rm(list = ls())

options(stringsAsFactors = F)

phe=read.csv(file="phe_.csv", header=T)

colnames(phe)

phe$futime_month=round(phe$futime/30,1)

library(survival)

library(survminer)

sfit <- survfit(Surv(phe$futime_month, phe$fustat)~phe$group, data=phe)

summary(sfit)

gg=ggsurvplot(sfit,

palette = c("orange","red","blue"),

risk.table =TRUE,

conf.int =F,

legend.title = "",

legend.labs = c("CD4CD103High/Grade3", "CD4CD103High/Grade1+2","CD4CD103Low/Grade1+2"),

xlab ="Time(months)",

break.x.by = 10,

risk.table.title="",

break.time.by = 10, #X轴间距，可换

risk.table.height=.25,

font.x = 14,

font.y =14,

font.legend=10,

risk.table.fontsize=6)

gg

**12. R Figure5C:**

rm(list = ls())

options(stringsAsFactors = F)

phe=read.csv(file="phe_.csv", header=T)

colnames(phe)

phe$futime_month=round(phe$futime/30,1)

library(survival)

library(survminer)

sfit <- survfit(Surv(phe$futime_month, phe$fustat)~phe$group, data=phe)

summary(sfit)

gg=ggsurvplot(sfit,

palette = c("orange","red","blue","sky blue"),

risk.table =TRUE,

conf.int =F,

legend.title = "",

legend.labs = c("CD8CD103High/Grade3", "CD8CD103High/Grade1+2","CD8CD103Low/Grade1+2","CD8CD103Low/Grade3"),

xlab ="Time(months)",

break.x.by = 10,

risk.table.title="",

break.time.by = 10, #X轴间距，可换

risk.table.height=.25,

font.x = 14,

font.y =14,

font.legend=10,

risk.table.fontsize=6)

gg
